# The Role of Worldviews in Predicting Support for Recreational Cannabis

**DOI:** 10.3389/fpsyg.2022.880537

**Published:** 2022-05-31

**Authors:** Gordon Sammut, Rebekah Mifsud, Noellie Brockdorff

**Affiliations:** ^1^Department of Criminology, University of Malta, Msida, Malta; ^2^Department of Cognitive Science, University of Malta, Msida, Malta

**Keywords:** worldviews, symbolic universes, social axioms, moral frameworks, deep stories, recreational cannabis, social representations

## Abstract

We studied the role of worldviews in the endorsement of proposals for the legalisation of recreational cannabis. Drawing on literature on generalised belief structures, we developed categorical measures for five worldviews drawing on commonalities in the typologies reviewed (Orthodox, Localised, Reward, Pragmatist, and Survivor). We proceeded to study the relative influence of worldviews in support of a range of items concerned with the legalisation of recreational cannabis amongst a randomly generated sample (*N* = 1000) in Malta. Our findings demonstrate that the Orthodox worldview stands in contrast to all others in opposing the proposals and constitutes the resistance group to legalisation. On the other hand, no other worldview unilaterally supports the proposals albeit these are, on an individual basis, favourably perceived. Our study further demonstrates that proportions of variance accounted for by the worldview measures we adopted are comparable to those exercised by demographic variables demonstrating significance. We propose that the study of worldviews is critical in understanding social and political alliances that come together to support or oppose particular politicised projects or collective courses of action.

## Introduction

The encounter of novel objects or events in social life requires interpretative representational work on the part of individuals to make the unfamiliar familiar (Moscovici, 2008). During the course of sharing perspectives in intersubjective communication, individuals develop construals (Ross and Nisbett, 1991) of novel items in their environment that make sense to them in light of their prior knowledge and experiences. This sense is at once individual and social. To the extent that it is communicable and fulfils certain precepts expected by others, we can refer to it as common sense (Sammut and Bauer, 2021). Such a conception of common sense immediately makes clear the fact that common sense is relative and particular to socio-historical epochs: what is common sense to one may be nonsense, or non-common sense, to others who inhabit a different social or historical setting. It follows, therefore, that some construals elaborated by particular social groups during a given time may be more or less common sensical than other construals circulating in the same public during the same time period. Social representations scholars have argued that knowledge is context bound and tied to its processes of production (Jovchelovitch, 2019). This criterion applies to common sense construals as much as it does to formal disciplinary knowledge. Consequently, it is pertinent to question how, in our current era, certain novel construals are positively appraised by some and at the same time abjectly rejected by others.

One issue that has been highly contested in various Western countries in recent years is the legalisation of recreational cannabis. Traditionally considered a gateway drug (Fergusson et al., 2006), the recent medicalisation of cannabis for the treatment of chronic pain and other ailments (Martín-Sánchez et al., 2009), along with the proliferation of cannabinoid products that report below the limit concentrations of tetrahydrocannabinol (THC), the main psychoactive ingredient in cannabis, have led to a revision of laws in many Western countries accompanied by strong debates regarding the justificatory basis for its criminalisation. Many countries saw the rise and proliferation of NGOs advancing the decriminalisation cause. Some countries, such as Malta, proceeded to introduce medical cannabis by prescription, dispensing it from regular pharmacies. Other countries, such as Portugal, decriminalised consumption and possession of cannabis for personal use. Yet other countries, such as the Netherlands, tolerate some forms of recreational use, typically within designated premises like “coffeeshops.” These various legal instruments imply representations of the drug and its users that are different from the traditional ‘junkie’ in search of the next high. On the one hand, some represent cannabis users as self-medicating patients seeking relief from debilitating conditions. Consequently, such users should not be stigmatised and punished through incarceration. Rather, they should be validated and assisted in obtaining relief in legitimate ways. On the other hand, some resist the victimisation implied in representing cannabis users as patients. They argue that prohibition saves few at the expense of the many and that individuals should be free to make their own choices as they do with other substances like alcohol and tobacco, given that most only ever use cannabis occasionally for recreational purposes without developing a habit. Both representations converge in enlisting sympathy over scorn for the cannabis user.

Given the current diversity of representations about cannabis and its use that are in circulation in contemporary Western societies, which provide in their turn justification for highly contrasting public policy measures, it is legitimate to question which social representation will stick and go on to shape laws and behaviours in the years to come (Bertoldo and Castro, 2018). Persuasion scholars have traditionally queried how messages can be designed for maximum appeal (Sammut and Bauer, 2021). The problem with this line of inquiry is that, as Billig (1996) notes, persuasion involves a rhetorical exercise in which an individual comes to agree with an interlocutor about a depiction of the object as presented. In the case of cannabis, certain representations are longstanding whilst others re-present the issue in novel ways (Buhagiar and Sammut, 2020). Consequently, perceivers are required to assimilate or accommodate new representations in their previously acquired repertoire (Sammut and Bauer, 2021). This is where the question of “stickiness” (Breakwell, 2014) becomes critical. In other words, which representation of cannabis will stick with whom, and for what purpose (Buhagiar and Sammut, 2020)? We propose that representations stick not as a function of characteristics of the social representation in itself (Breakwell, 2014). Rather, representations stick as a function of their resonance with particular worldviews that individuals already inhabit. That is, if the sense imparted by a novel social representation makes resonates with the worldview inhabited, then the representation is positively appraised and sticks with that individual. Conversely, to the extent that it does not resonate, it fails to stick. For instance, in a hedonistic worldview, the proposal to legalise medical cannabis as a treatment for chronic pain sticks by virtue of the fact that cannabis removes pain – a contrasting stimulus to the worldview. However, in a religious worldview that places a value on piety, getting high does not make sense as it leads to loose behavior. In this case, the representation of cannabis as a ‘recreational’ pursuit does not stick.

In this paper we investigate whether support or resistance toward recreational cannabis is a function of an individual’s worldview. We define worldviews as generalised outlooks on life and on one’s place in the world that provide a general template for social conduct. We propose that worldviews serve to interpret social stimuli in characteristic ways and, as a result, to identify opportunities and threats in the social order (Koltko-Rivera, 2004). We argue that worldviews form the basis of resonance between individuals holding their own personal perspectives. In this way, they provide the basis for the establishment of social relations. We contend that individuals with similar worldviews perceive and interpret the world, the scope of life and stimuli in their environment in similar ways. Individuals with similar worldviews hold perspectives that are able to inter-penetrate (Sammut, 2011) and the construals they fashion individually are mutually sensible (i.e., appeal to a *common* sense) (Sammut and Bauer, 2021). Conversely, to the extent that worldviews between interacting individuals are at odds, they result in perceived opportunities or threats that transpire as unrealistic, illogical or insensible (i.e., nonsensical) to the other party. We argue that worldviews provide thus a bedrock for the establishment of common sense. To the extent that individuals perceive elements in their environment in the same way according to a shared worldview, the personal sense they make and communicate to each other will be understandable, legitimate, and reasonable. In other words, *common* sense. To others with a different worldview, that perspective may transpire as *nonsense* (Sammut and Bauer, 2021). It is recognised as a personal perspective, but one that is wrong, faulty or unreasonable. What is an opportunity to some is a threat to others. Consequently, one would expect that individuals with similar worldviews will hold similar attitudes toward novel objects in their environment, as this is appraised similarly by those who share the worldview and differently from other worldviews that construe it differently. Worldviews thus provide a mechanism for forging alliances in the social domain in pursuit of particular ends that fulfil descriptive and prescriptive precepts of the world and how it operates. In this paper, we explore the implications of holding particular worldviews on attitudes supporting or proposing the legalisation of recreational cannabis. We proceed with a review of the literature on worldviews and their varied conception before presenting findings from a study that investigates their role in supporting policies regarding the legalisation of recreational cannabis. We conclude by demonstrating how the *Orthodox* worldview differs from various others in substantive ways and constitutes the resistance approach to changes in policy that threaten the current order.

## Worldviews

A number of theories in the social sciences have purportedly identified a range of universal immutable precepts that shape and guide human action in the real world. Notably, four theories originating in different strands of social science report a range of five variants with remarkably similar properties. We proceed to review these hereunder. It is worth noting that our conception of worldviews rests on categorical rather than dimensional criteria. Koltko-Rivera (2004) provides a comprehensive review of the latter but does not engage with the former. In the present study, however, we were interested to identify (a) which and (b) to what extent particular worldviews and not others served to support or resist particular proposals for policy. In Koltko-Rivera’s dimensional approach, individuals will demonstrate variable tendencies toward a range of concurrent worldviews. Some worldviews will be more strongly endorsed than others, but individuals are not held to operate within the confines of specific worldviews. This may or may not be right. By analogy, one could argue that the traits of masculinity and femininity may be measured across individuals, regardless of whether they are male or female. You can have males who are high in femininity as well as females who are high in masculinity, along with the traditional masculine males and feminine females. However, in certain instances, the categorical variable of gender might be more interesting than the dimensional variable of masculinity/femininity. With the exception of gender-fluid individuals, many experience their gender in exclusively categorical terms, and this may in itself be useful for psychological inquiry. In the same way, whilst we agree with Koltko-Rivera (2004) that any worldview typology rests on a set of underlying dimensions, we wanted to examine the relevance of inquiring into worldview typologies directly given that an individual cannot ‘inhabit’ distinct and opposing worldviews simultaneously as these may, in certain circumstances, prove contradictory. For instance, somebody who perceives a stimulus as positive due to the fact that it provides an opportunity for change cannot, *at the same time* and *in the same manner*, perceive it equally in negative terms as a threat to social order. The stimulus will be an opportunity for some and a threat to others depending on how the individuals orientate themselves to the social order and whether they see cause in pursuing change or otherwise. But they cannot both wish for stability and change. Moreover, we also wanted to empirically examine the extent of influence exercised by singular worldviews on a material change of public policy. Our worldview model, therefore, adds theoretical and empirical depth to the psychology of worldviews (Koltko-Rivera, 2004) by examining their social psychological implications on support or resistance of policy.

### Symbolic Universes

In their study of European publics, Salvatore et al. (2018) report a range of five *symbolic universes* variably diffused throughout national jurisdictions in the European Union. Salvatore et al.’s (2018) study is concerned with dynamics of sense-making, that is, processes of interpretation of the world that shape experience. The authors examined reactions to the financial crisis of 2008 and the immigration crisis of 2015 in Europe. According to Salvatore et al. (2018), the interpretation of both these crises by Europeans were guided by generalised, affect-laden meanings embedded in cultural descriptions of the world and how it works. These meanings, in turn, channel the particular opinions individuals develop of happenings in their environment. The authors refer to these generalised meanings as symbolic universes to denote an entire and self-contained field of experience rather than any particular element within it or any dimension underlying it (Koltko-Rivera, 2004). We agree with Salvatore et al.’s (2018) conclusions and our measure of worldviews is operationalised in such categorical terms. Salvatore et al. (2018) go on to claim that an individual’s experiencing of the world is guided and shaped by the symbolic universe the individual as a sense-maker identifies with. Collectively, symbolic universes give rise to discourses about European unity and identity that spread over different domains of institutional life and across different segments of the population. According to the authors, symbolic universes constitute worldviews that describe how people frame their worlds. Each symbolic universe presents a particular interpretation of the same social order, emphasizing certain aspects and de-emphasizing others.

The *Ordered* universe is characterised by a generally positive attitude toward the world and its people and institutions as well as identification with transcendent values and ideals that places a high priority on ethics, righteousness, and morals. The *Interpersonal bond* universe is marked by a positive, optimistic vision of the world rooted in deeply emotive and affiliative bonds where belonging is an end in itself. The *Niche of belonging* universe shares a focus on affiliation coupled with, however, a negative connotation of the world outside one’s primary network. In this universe, affiliation provides security against a threatening social order. The *Caring society* universe is characterised by a vision of society as trustworthy provider of common goods and services that provides a positive vision of the future and an obligation to abide by social norms. The *Others’ world* universe is marked by a desperate vision of the world that is perceived as untrustworthy. In this universe, individuals are powerless and face a constant struggle for survival with damage limitation as the only realistic aspiration.

Salvatore et al. (2018) go on to argue that symbolic universes not only serve to make sense of one’s environment on an individual basis but that these generalised worldviews serve to establish social relations with like-minded others. The authors claim that the Ordered and Caring Society universes appeal to axiological beliefs that transcend immanent affiliative bonds and that therefore serve in the creation of bridging social capital (Sammut, 2011) across particular communities. Conversely, the Interpersonal Bond and Niche of Belonging universe focus on relational networks and consequently foster bonding forms of social capital. Finally, the Others’ World universe constitutes an anomic outlook that represents the primordial struggle for survival. The authors conclude by proposing that the study of symbolic universes is highly revealing both in terms of understanding individual inclinations as well as in gaining insight into societal tendencies in light of the universes’ relative distribution across national jurisdictions.

### Social Axioms

Leung and Bond’s (2009) extensive cross-cultural inquiry provides a five-factor typology of *social axioms*. These are domain-general, context-free beliefs that function as generalised expectancies of how the personal, social, environmental and spiritual worlds function and on the basis of which individuals make predictions about the outcomes of their actions. Social axioms serve to anticipate, guide and rationalize behaviour such that individuals can manage their lives more effectively, realize their goals and avoid undesirable outcomes (Boehnke, 2009). Leung and Bond (2009) ascribe evolutionary roots to social axioms. They argue that individuals must necessarily develop ways to simplify the processing of information that emanates from their environment if they are not to be overwhelmed by the massive sensory data at their disposal. They thus develop tendencies to select the stimuli that seem most important to them in their lives, chunking stimuli together in bundles and processing them as if they were one entity. These generalised chunks of belief simplify information processing by representing people’s cognitive map of the social world, directing experiencing in determined interpretative dimensions.

The authors identify five cross-cultural axiomatic maps, where ideas related to religion, fate, complexity, reward and cynicism hang together (Boehnke, 2009; Leung and Bond, 2009). In *religiosity*, individuals identify with the existence of a supernatural being and endorse a number of beliefs about the beneficial social functions of religious institutions and practices. This reflects an image of conservative and conventional individuals, strongly oriented to socially accepted norms who strive to be empathic and tender-minded in relating with others. In *fate control*, individuals subscribe to beliefs suggesting predetermination of events by external forces coupled with the ability for people to influence the negative impact of such forces. This reflects an image of controlled and balanced individuals with a warm and empathic disposition who also hold a negative work orientation and who score high on active impression management. In *social complexity*, individuals hold that there are multiple ways to solve problems and that people’s behaviour varies across situations. This social axiom reflects endorsement of self-management, a nurturing and open-minded attitude and a temperament that is attuned to the needs of others. In *reward for application*, individuals assert that investment of effort, knowledge and careful planning lead to positive outcomes. The typical individual ascribing to this axiom is a hard-working person motivated by the desire to achieve a higher social status but also socially pleasant and adherent to social norms. According to Boehnke (2009), such individuals tend to be assertive, socially skilled, and optimistic, as well as somewhat neutral with regards to understanding or nurturing others’ feelings. In *social cynicism*, a negative view of human nature prevails marked by outgroup bias, a mistrust of social institutions and a belief that people tend to pursue goals using unethical means. The social image of a typical social cynic is that of a rather unsociable person with low social skills and strong opinions who demonstrates low self-control and disregard for other people’s impressions (Boehnke, 2009).

Leung and Bond (2009) go on to argue that social axioms serve to orient and guide individuals in an infinitely complex social world. Thus, we expect those who emphasize religiosity to pursue supernatural interventions, whilst those who devalue it can be expected to provide more mundane explanations of events. Similarly, those high in fate control expect external events to influence their lives, those high in complexity to disagree with simple explanations, and those who emphasize beneficial outcomes to look out for investment opportunities in managing resources. Finally, we can expect those high in social cynicism to view events through a cynic’s lens. In this way, axioms construe events in a certain way and guide human engagement with the world accordingly.

### Moral Frameworks

Haidt (2012) popularised the concept of moral frameworks in a volume exploring the roots of righteousness. He starts by arguing that moral reasoning is part of the human characteristic to relate socially. When human beings become aware of the actions of others, they react morally by exerting collective judgement on the act, which might or might not benefit the individual. Consequently, moral reasoning is not subjective. Rather, it involves claims about what others might have done wrong. Individuals are not punished because somebody else does not like what they do. They are punished by reference to a common standard that lies outside anybody’s subjective preferences. Moreover, moral reasoning serves not to justify why we ourselves come to a particular judgement. We do moral reasoning to convince others to join us in our avowed judgement. In other words, moral reasoning appeals to common sense (Sammut and Bauer, 2021). Like Leung and Bond (2009) and Haidt (2012) goes on to argue that this common sense has roots in our genetic evolution and contains a number of characteristic immutable elements. For Haidt, life presents a series of opportunities that, in the case of humans, are maximised when mutually beneficial. Collaboration enlarges the pie that we ultimately share. Collaboration, therefore, has been hard-wired into our moral reasoning and emotive baggage in a way that inclines us to mutually beneficial social relations. As a result, we feel pleasure when people cooperate with us and sadness or disgust when cheated. Haidt argues that humans have evolved specific perceptive abilities, like mental receptors, that trigger particular emotions and are accompanied by specific moral concerns. These have evolved in response to particular phylogenetic challenges our ancestors faced as a species. In this way, we are able to react intuitively to particular happenings in our social environment. These form the basis of the moral frameworks we share as a species and which we use to navigate social life.

Haidt (2012) claims that five adaptive challenges stood out in our ancestral environment: (i) caring for vulnerable children, (ii) forming partnerships with non-kin to benefit from reciprocity, (iii) forming coalitions to compete with other coalitions, (iv) negotiating status hierarchies, and (v) keeping free from parasites and pathogens that spread quickly when people live in close proximity (p. 139). These adaptive challenges were overcome by the evolution of a moral framework in our cognitive arsenal that enabled the species to progress from one to the next in the evolutionary ladder in step-wise fashion. We are thus equipped with five *moral frameworks* concerned with (i) care/harm, (ii) fairness/cheating, (iii) loyalty/betrayal, (iv) authority/subversion, and (v) sanctity/degradation. The *Care* framework, according to Haidt, evolved in response to the need to care for vulnerable children. This makes us sensitive to others’ needs and sufferings and make us despise cruelty and inclined to care. The *Fairness* framework evolved in response to the challenge of cooperating without getting exploited and makes us sensitive to acts of reciprocal altruism and an inclination to punish those who do not deserve their dues. The *Loyalty* framework evolved in response to the need of forming coalitions. This makes us sensitive to the fact that some might not be with our team and makes us ostracize those who betray us (i.e., social identity and the ‘black sheep’ effect) (Sammut and Sartawi, 2012). The *Authority* framework evolved in response to the challenge of living in social groups. It makes us sensitive to rank and reputation and inclined to identify and scorn those who act out of line. Finally, the *Sanctity* framework evolved in response to the challenge of living in a communal world that is prey to pathogens and parasites. It constitutes a behavioural immune system which makes us wary of various objects and threats and which enables people to invest in objects and events that are important for binding groups together (p. 170).

Haidt goes on to argue that the evolution of these frameworks proceeded in probable steps. The first was the evolution of social instincts. In ancient times, Haidt argues, loners would have been more likely to fall prey to predators than more gregarious siblings who preferred to wander around in groups. Consequently, the ones equipped with social instincts were more likely to survive and lonely tendencies to be weeded out of the species. Secondly, those who then showed a tendency for helping others would have been more likely to get help themselves when in need and, therefore, to survive better than their selfish counterparts. This would have led to the formation of social groups where humans banded for security purposes. In such a context, rank and reputation would have come to the fore as people were called upon to judge the actions of others within the same group and the extent to which one’s tendencies sustained or betrayed collective aspirations. Those who lacked a sense of shame or love of adulation at this stage would have been less successful than their more assuaging counterparts. Finally, the rise of the ability to treat duties and principles as sacred would have established the community beyond the individual lifespan of its members and leaders. Those members disinclined to collective transgenerational pursuits who satisfied themselves with their lot in the present would have lost out to their counterparts over a number of generations as private wealth accumulated across time.

### Deep Stories

Hochschild (2016) reports findings of an investigation with Tea Party Movement members in the American South. She starts her investigation admitting a certain difficulty in understanding these points of view as it seemed, from the outside, that these points of view were somewhat self-defeating. They seemed to support the policies of a Republican party whose resistance to social support programmes would seem to hurt Republican party strongholds in Louisiana more than any other location. Rather than dismissing these views as ignorance (Sammut and Sartawi, 2012; Sammut, 2019), Hochschild spent a period of time conducting ethnographic work in these jurisdictions to understand better how these political positions were justified by their supporters. In the process, she identified five *deep stories* that lend validity, in their particular ways, to Tea Party support.

The first deep story described by Hochschild is *The Team Loyalist*, who values loyalty above all else. The Team Loyalist is motivated by accomplishing a team goal and is ready to work hard in line with a larger moral code that sees some ahead of them and some behind them in pursuit of the American Dream. Hard work is important because it confers honor. Anyone aided by social movements to jump the line does not share this value and abides by a looser moral code. The Team Loyalist adopts a whole company perspective and understands their place in the grand scheme of things. They refrain from the pursuit of individual gains that disrupt the system and make it less efficient for all. For this reason, the Team Loyalist has little sympathy for those claiming welfare – they should bite the bullet and get a job. If they do not, they only have themselves to blame for their condition. Children who start off disadvantaged can be churched into this spirit and as they grow they will be able to work their way up in life. The Team Player believes that we all “have to find our own niche and learn to be happy where we are” (p. 161). In the process, one might have to brave some issues and be accommodating, so that the system could function. Sometimes one just “had to suck it up and just cope” (p. 163). In return, the Team Loyalist sports an endurance self that is busily rooted in a stable network of family and friends.

The second deep story is that of *The Worshipper*, who demonstrates an invisible but meaningful renunciation to a transcendental entity. What one has, even if that is a lot, one owes to the grace of a higher being. And what one might wish for may at times be one’s own undoing. It is therefore best to leave matters to the grace of god, who might ultimately grant one’s wishes in mysterious ways. The fundamental truth about life is that everything is transitory, only god is permanent. One just need to have faith and let god do the work. This is not easy, as we are fallible humans prone to envy and greed. But with god’s grace one can succeed in doing the right thing and when we do, god will give us plenty.

The third story described by Hochschild is *The Cowboy*, who demonstrates a fearless stoicism in the face of adversity. The Cowboy is daring, and good things happen to people when they dare to take risks. The Cowboy accepts the fact that in so doing, they will have to take some knocks. We all make our own decisions and we must live with the consequences, even when they are unpleasant. When things turn out badly, one needs to be strong and endure. This does not make one a victim; it makes one brave in the face of adversity.

A fourth story identified by Hochschild is that of *The Rebel*, described as a team loyalist with a cause. The Rebel has a number of characteristics that are shared with other stories. In particular, though, The Rebel is identified in being an activist; not enduring but fighting to make things better. The Rebel is a fighter but not a Cowboy, religious but not a Worshipper and a Team Player but critical.

The fifth deep story Hochschild alludes to but one that is not directly observed in her work is *The Cosmopolitan*. This refers to an uprooted, loosely attached self that is not grounded in an immediate community, that is prepared to know a lot of people just a little bit rather than the other way around, and that presents a mobile, migratory self in search of favourable opportunities. From the outside it looked like this story subscribed to an ‘anything goes’ mentality but The Cosmopolitan pride themself in exposure to diverse sets of moral codes.

It is worth noting that Hochschild’s deep stories show many interlinkages and similarities with each other. Certain characteristics are shared but each story retains an overarching style that justifies particular courses of action or inaction in the face of a common event. All of these stories supported the American Tea Party, for different reasons, and made sense of it in their own ways. Even across stories, alliances were forged as a result of a common antipathy to federal regulation, for instance. Yet different stories construed the world in different ways and as a result advocated and pursued different remedies to similar ails.

### Common Features

At this point, we would like to argue that the concepts reviewed above demonstrate significant overlap between them. We believe this is due to the fact that they each tap the very same phenomenon, that is, that of a generalised outlook on the world and the life that takes place within it. The convergence is reminiscent of the Indian parable of an elephant groped by various blind men where one highlights a certain feature and somebody else another, all thinking they are describing a different animal but in reality each describes a particular characteristic of the same entity. We contend that symbolic universes, social axioms, moral frameworks and deep stories are, despite their overt differences, functionally equivalent and respectively attuned to different features of the same phenomenon (see Table 1). For instance, what Salvatore et al. (2018) identify as Ordered universe, emphasizing ethics, righteousness, and morals based on transcendent values and ideals, Leung and Bond (2009) identify as the Religiosity axiom that enables identification with a supernatural entity. Haidt (2012) similarly proposes a Sanctity framework, by which individuals hold some elements in their environment as sacred. And Hochschild (2016) identifies a Worshipper story in which individuals subjugate to a transcendental entity. We argue that these are elements of an *Orthodox* worldview and we proceed to similarly tie a thread across the others as per Table 1.

**TABLE 1:** Syncretic conceptualisation of worldviews.

*Worldviews*	Symbolic universes	Social axioms	Moral frameworks	Deep stories
*Localised*	Interpersonal bond	Social complexity	Loyalty/betrayal	Team player
*Pragmatist*	Niche of belonging	Fate control	Fairness/cheating	Rebel with a cause
*Orthodox*	Ordered universe	Religiosity	Sanctity/degradation	Worshipper
*Reward*	Caring society	Reward for application	Care/harm	Cosmopolitan
*Survivor*	Others’ world	Social cynicism	Authority/subversion	Cowboy

In summary, it seems that human cognition has evolved a capacity for grand outlooks (i.e., worldviews) that confer meaning to human existence and enable action in pursuit of goals that are sensible enough to like-minded others to enable joint action, even in the face of adversity. In this way, we believe human beings have been able to overcome setbacks and thrive, by reconstructing meaning according to a revision of the story from one to another, like changing gears in a moving vehicle to adapt to a changing terrain (Ciavolino et al., 2017). We contend that this constitutes the essence of human adaptability to situational challenges, that is, the ability to revise one worldview for another and move on by forging new alliances rather than give up and perish. Consequently, whilst personal dispositions (e.g., attitudes) may incline an individual favourably or unfavourably toward a certain object, the embedding of these inclinations in generalised worldviews enables also the establishment of a common sense, as we discussed above, that transcends the particular attitude and that enlists one with similar others in grand projects that purport to advance human action in one direction and not another. In this way, worldviews serve essentially to forge together coalitions for action that compete with other, differently engaged coalitions (Buhagiar and Sammut, 2020). Our conception of worldviews is similar to Koltko-Rivera (2004) definition emphasising beliefs and assumptions about life and reality that are used as an interpretative lens to understand reality and one’s place in it, and that has both ontological (what is and what ought to be) and epistemological (what is known, what can be known and how it can be known) dimensions (p. 3–4). For this reason, we studied whether distinct worldviews lent support or rejected proposals for the legalisation of recreational cannabis in characteristic ways, in an effort to understand alignments in the social order in reaction to policy proposals. We proceed with reviewing the methodological details of the study before turning to a presentation of findings and a discussion of their implications.

## Materials and Methods

### Aims

We wanted to investigate whether worldviews exercised an influence on attitudes toward the legalisation of recreational cannabis. We expected relatively conservative worldviews to demonstrate a negative attitude toward these proposals whilst relatively liberal and optimistic worldviews to be more accepting. Consequently, we posed the following hypotheses:

H1: We expected all five worldviews to individually exercise a significant net effect on attitudes toward the proposals, either supporting the proposals as an opportunity to strengthen their worldview, or rejecting them on the basis of a threat to their worldview.

H2: Specifically, we expected that Reward and Localised worldviews would demonstrate significantly more positive attitudes toward the legalisation of recreational cannabis than Orthodox and Pragmatist. We reasoned that the Reward worldview would see an opportunity in the proposals, for profit or for pleasure, and that the Localised worldview would be sympathetic with those who wish to consume cannabis and opt to not get in the way. On the other hand, we reasoned that the Orthodox worldview would perceive recreational cannabis as a threat to the prevailing order and a detraction from higher ideals. We also reasoned that the Pragmatist worldview would perceive this as an unnecessary perturbation that will require adjustment to a state of affairs that benefits others. We therefore expected the means endorsing the attitudinal items pertaining to the former worldviews to differ significantly from the means endorsing the attitudinal items pertaining to the latter.

Both hypotheses were tested using a sequence of hierarchical linear regression tests for the various attitudinal items which included a bloc controlling for socio-demographic differences and another bloc incorporating the various worldview categories to determine their net effect (H1) as well as mean differences between them (H2). Results were analysed relative to the Localised worldview.

### Participants and Procedure

We carried out a nationwide survey amongst a random sample of 1000 respondents resident in Malta. 481 respondents identified as male with the remaining 516 identifying as female. Respondents were over the age of 18 (*M* = 45.04, SD = 16.98) and resided across various districts in Malta and Gozo. 51 respondents reported a primary level of education, 414 a secondary level, 211 reported a post-secondary level and another 319 reported a tertiary level of education. Respondents were recruited through a random telephone number generator and the questionnaire was administered during a phone call held at a time and date of the respondent’s choosing. Interviews were carried out between March and June 2021 and lasted an average of 50 min. No personal identifiable data was gathered for the purpose of this study. The questionnaire was available in both English and Maltese, but all respondents opted to answer the Maltese version. We subjected the study to self-assessed ethical clearance following the University of Malta’s research code of ethics and ethical clearance procedures.

### Instruments

The study formed part of a wider project investigating the Maltese public’s perceptions of the police and issues related to criminal behaviour and matters of security. The questionnaire used was divided into eight sections. The first gathered demographic data about respondents as well as a measure of their worldviews, the second pertained to perceptions of security, the third concerned attitudes toward immigrants, the fourth section was concerned with fear of crime, the fifth and sixth with general policing and community policing respectively, the seventh concerned societal debates concerning criminalised behaviours, and the eighth provided respondents with a measure of Maltese identity. For the purpose of this paper, we analyse data gathered from sections one and seven as follows.

To elicit respondents’ worldviews, we presented them with a series of five vignettes (Table 2) designed by the authors incorporating a mix of items solicited from publicly available measures for symbolic universes (Salvatore et al., 2018), social axioms (Leung and Bond, 2009), and moral frameworks^1^. In view of the fact that the four worldview theories reviewed above all note similarities between worldviews, we decided against the use of discrete items due to the fact that single items would thus be expected to load onto more than a single worldview. The study of the constellation of items to extract five worldview factors was anticipated to be sufficiently cumbersome to preclude study of the relations between worldviews themselves and other variables they might influence, as per the present study. Consequently, the presentation to respondents of vignettes that incorporated various items faithful to the various worldview descriptions provided in the literature as a gestalt was deemed preferable in view of its more efficient administration. Following the reading out of each vignette, we asked respondents to indicate the extent of their endorsement of the worldview on a Likert scale from 1 (Strongly Disagree) to 5 (Strongly Agree). After all vignettes were presented to respondents, they were asked to select the one that approximated their views most. In this way, we ensured that respondents cognitively processed each vignette in sufficient depth to rate their endorsement of it, whilst providing respondents an opportunity to self-categorize according to their worldview.

**TABLE 2:** Worldview measures.

Worldview	Vignette
Localised	The future depends on us and the choices we make. Every problem has a solution. Each and every one of us can make an effort to fix the laws and institutions so that they can be just and equal for everyone. Like this we can better address the needs of people and society.
Pragmatist	In life we must adapt ourselves to our circumstances and sometimes we need to go with the flow in order to avoid trouble. The rich and powerful protect their own interests, whereas the kind-hearted suffer. Sometimes you have to work around the rules to help your loved ones.
Orthodox	To succeed in life, we need to follow the rules and local customs in order to maintain social order. We also need to show respect to each other and carry out our duties. Like this we can help others in our community.
Reward	In life, you get what you deserve. Life’s challenges are overcome with the efforts we make, and these may offer new opportunities. One must co-operate with others, respect authority, and carry out one’s duties. Our efforts will eventually lead to success.
Survivor	In life, things rarely end up well. People are what they are, and good people usually suffer and are exploited. It is best for one to keep his/her head down and get on with it.

To elicit respondents’ attitudes toward proposals concerning the legalisation of recreational cannabis, we presented them with four items (Table 3) sourced from public discussions concerning the issue. A public consultation exercise was undertaken by the Maltese government following the publication of a White Paper in March 2021 which advanced a number of legislative proposals. These were widely debated at the time both online and in regular media. The consultation exercise took place during the same time period as the undertaking of this survey and was terminated in May 2021. We asked respondents to rate their agreement with each of the items we presented to them on a Likert scale from 1 (Strongly Disagree) to 5 (Strongly Agree). High scores on these measures indicated favourable attitudes toward legalisation whilst comparatively lower scores indicated disagreement.

**TABLE 3:** Items debating the legalisation of recreational cannabis in Malta.

1. The use of cannabis is very common, so cannabis should be legalised to avoid criminalisation.
2. The majority of people who use cannabis do not go on to become addicted to more powerful drugs.
3. People who use cannabis regularly do so to improve their quality of life.
4. Instead of fighting the use of cannabis, the state should focus more on helping those in need.

Categorical demographic data was gathered for Age (16–25; 26–35; 36–45; 46–55; 56–65; 66+), Gender (Male, Female, Other), Educational Attainment (Primary; Secondary; Post-Secondary; Tertiary), District of Residence (Gozo; Northern; Northern Harbour; Southern Harbour; South Eastern; Western), Relationship Status (Widowed; Not Married; Married; Divorced/Separated/Annulled) and Occupational Status (Employed; Unemployed; Student; Homemaker; Retired).

We created dummy variables for each of the worldviews (as per Table 2) for the purpose of data analysis. In hierarchical regression, we inputted in the first block the demographic variables identified as exerting significant effects on the individual items. All these first models were necessarily statistically significant in the regression output. To examine the isolated effects of worldviews, we proceeded to include all the dummy coded worldviews in the second block of the hierarchical linear regression models. For the purposes of this study, we were primarily interested in the results of the regression output including both blocks. Collinearity diagnostics performed on the various models presented no concerns.

## Findings

We asked respondents to self-categorise their worldviews after rating a sequence of five vignettes corresponding with each worldview respectively (Table 2). Of the total of valid responses on this measure (*N* = 978), 26.9% (*n* = 263) self-categorised as *Localised*, 21.1% (*n* = 206) as *Orthodox*, 20.4% (*n* = 200) as *Survivor*, 20.2% (*n* = 198) as *Reward* and 11.3% (*n* = 111) as *Pragmatist*. We also asked respondents to rate the extent of their agreement with four items representing views concerning recreational cannabis. Respondents tended to agree with the claim (i) that legalisation is warranted due to the fact that cannabis use is pervasive ($\bar{X}$ = 3.32, SD = 1.29). They were neutral with regards to the claim (ii) that cannabis is a gateway drug ($\bar{X}$ = 3.00, SD = 1.17). Conversely, respondents disagreed with the claim (iii) that people who use cannabis do so to improve their quality of life ($\bar{X}$ = 2.88, SD = 1.17). Finally, they expressed themselves in favor of the claim (iv) that the state should focus on helping those in need instead of fighting the use of cannabis ($\bar{X}$ = 3.85, SD = 1.06).

We proceeded to test for demographic differences in the endorsement of each of these claims using a series of one-way ANOVAs to examine differences in (a) Age, (b) Gender, (c) District, (d) Education, (e) Relationship Status, and (f) Occupational Status categories (Table 4). This procedure was intended to eliminate demographic variables that did not lead to statistically significant differences in responses to the cannabis items. With regards to the claim: *The use of cannabis is very common, so cannabis should be legalised to avoid criminalisation*, we found statistically significant differences in Age [*F*(5,972) = 8.624, *p* < 0.01], Relationship Status [*F*(3,964) = 5.938, *p* < 0.01] and Occupational Status [*F*(4,967) = 9.495, *p* < 0.01]. With regards to the claim: *The majority of people who use cannabis do not go on to become addicted to more powerful drugs*, we found statistically significant differences between categories for Age [*F*(5,968) = 10.119, *p* < 0.01], Gender [*F*(1,969) = 5.321, *p* < 0.05], Education [*F*(3,965) = 5.142, *p* < 0.01], Relationship Status [*F*(3,960) = 9.357, *p* < 0.01] and Occupational Status [*F*(4,963) = 5.027, *p* < 0.01]. We found statistically significant differences in response to the statement: *People who use cannabis regularly do so to improve their quality of life;* there were differences in Age [*F*(5,972) = 12.465, *p* < 0.01], Gender [*F*(1,973) = 17.833, *p* < 0.01], Relationship Status [*F*(3,964) = 9.074, *p* < 0.01] and Occupational Status [*F*(4,967) = 7.755, *p* < 0.01]. For responses to the final item, *Instead of fighting the use of cannabis, the state should focus more on helping those in need*, statistically significant differences transpired between categories for Age [*F*(5,971) = 3.311, *p* < 0.01] and District [*F*(5,971) = 7.180, *p* < 0.01]. Demographic differences were found, therefore, in response to each item we measured. Age was the only demographic variable to exert a significant difference on every item.

**TABLE 4:** Demographic data.

Variable	Category	Frequency
Age	16–25	151
	26–35	203
	36–45	191
	46–55	149
	56–65	127
	66+	179
Gender	Female	516
	Male	481
District	Gozo	82
	Northern	130
	Northern harbour	259
	South eastern	204
	Southern harbour	191
	Western	134
Education	Primary	51
	Secondary	414
	Post-secondary	211
	Tertiary	319
Relationship status	Widow/er	37
	Not narried	310
	Married/cohabiting	556
	Separated/divorced/annulled	87
Occupational status	Employed	595
	Unemployed	20
	Student	66
	Homemaker	169
	Retired	144

Given the fact that our study was concerned with the effect of worldviews on opinions regarding the legalisation of recreational cannabis (Table 5), we proceeded to run a series of hierarchical multiple linear regressions controlling for the demographic influences identified above, to test our two hypotheses. These enabled us to isolate the effect of worldviews on opinions (H1) and compare them relative to each other (H2) whilst controlling for socio-demographic influences.

**TABLE 5:** Descriptive statistics for each worldview.

Statements	Localised		Pragmatist		Orthodox		Reward		Survivor	
	*M*	SD	*M*	SD	*M*	SD	*M*	SD	*M*	SD
Recreational cannabis use is pervasive	3.32	1.34	3.37	1.21	3.07	1.26	3.44	1.29	3.42	1.31
Majority of users do not get addicted	3.10	1.14	3.17	1.15	2.74	1.14	3.03	1.19	3.02	1.22
Recreational cannabis improves quality of life	3.02	1.15	2.89	1.09	2.59	1.03	3.03	1.27	2.77	1.25
The state should focus on helping those in need	3.94	0.97	3.83	1.00	3.62	1.07	3.85	1.15	4.01	1.09

For the first item (recreational cannabis use is pervasive), the regression model was significant [*F*(7,940) = 7.065, *p* < 0.01], explaining 5% of the variance. The worldview measures on their own accounted for 1.1% of this. However, relative to the Localised worldview, none of the other worldviews considered contributed significantly to the model (Table 6).

**TABLE 6:** Predictors for recreational cannabis use is pervasive.

Worldview	*t*	Significance
(Constant)	21.096	<0.01
Pragmatist	0.388	0.698
Orthodox	–1.604	0.109
Reward	1.055	0.292
Survivor	1.683	0.93

For the second item (majority of users do not get addicted), the model was significant [*F*(9,928) = 6.269, *p* < 0.01] accounting for 4.8% of the variance of which 1.4% was attributable to worldviews. In this model, the Orthodox worldview was identified as a significant predictor relative to the Localised worldview (Table 7).

**TABLE 7:** Predictors for majority of users do not get addicted.

Worldview	*t*	Significance
(Constant)	14.450	<0.01
Pragmatist	0.245	0.806
Orthodox	–3.140	<0.01
Reward	–0.862	0.389
Survivor	–0.068	0.946

For the third item (recreational cannabis improves quality of life), the model was also significant [*F*(8,937) = 11.554, *p* < 0.01] and accounted for 9% of the total variance observed, of which 1.8% was attributable to worldviews. Once again, the Orthodox worldview emerges as a significant predictor relative to Localised (Table 8).

**TABLE 8:** Predictors for recreational cannabis improves quality of life.

Worldview	*t*	Significance
(Constant)	22.803	<0.01
Pragmatist	–1.233	0.218
Orthodox	–3.962	<0.01
Reward	–0.306	0.760
Survivor	–1.401	0.162

Finally, for the fourth item (the state should focus on helping those in need), once again the model was significant [*F*(6,949) = 3.173, *p* < 0.01], accounting for 2% of the variance, of which worldviews alone accounted for 1.6%. As for the previous items, the Orthodox worldview alone emerged as a significant predictor relative to Localised (Table 9).

**TABLE 9:** Predictors for the state should focus on helping those in need.

Worldview	*t*	Significance
(Constant)	28.462	<0.01
Pragmatist	–0.878	0.380
Orthodox	–3.261	<0.01
Reward	–0.863	0.388
Survivor	0.492	0.623

To sum up, our findings were only partially supported. Our first hypothesis that all worldviews would exert a significant net effect on the attitudinal items was supported for the Orthodox worldview in three out of the four items we studied. Moreover, our second hypothesis regarding the relative alignment of the worldviews with regards to the attitudinal items was supported only for the Orthodox worldview. Relative to the other worldview, the Orthodox worldview stood clearly in opposition to the proposals, but other worldviews which did not position themselves for or against in clear and unequivocal terms.

## Discussion

We studied the role of worldviews in predicting support for recreational cannabis. The legalisation of recreational cannabis has proven to be a contentious issue in many countries worldwide. Colloquially, some fear the repercussions on individuals, such as increased dependency, as well as associated societal repercussions, such as proliferation of coffee shops and petty theft. Many others, however, argue that cannabis is essentially a cure that promises relief from unnecessary ails and that has fewer negative effects than other substances already in widespread use, such as alcohol and nicotine. Either way, the legalisation of recreational cannabis stands to precipitate various changes in social habits by legitimating the practice of getting stoned. Consequently, it stands to bring about foreseen and unforeseen changes to the social order. In particular, cannabis users will not only not be penalised nor criminalised, they also stand to not be victimised for their actions either. Such a change in laws understandably appeals to some who identify or empathise with the cause. Others, equally understandably, would find the prospect abhorrent. It is therefore reasonable to expect that worldviews, in terms of the social order they imply and pursue, should play a role in the endorsement or otherwise of these proposals. Equally, as Koltko-Rivera (2004) has previously argued, worldviews should help reveal factions and demarcate supporters from opponents.

We started by reviewing the social science literature that has conceptualised worldviews in categorical ways. We noted stark similarities between symbolic universes (Salvatore et al., 2018), social axioms (Leung and Bond, 2009), moral frameworks (Haidt, 2012), and deep stories (Hochschild, 2016). All propose a typology of five variants. Loosely and in no particular order, the first is predominantly concerned with interpersonal relationships (Localised), the second with a prevalent transcendental social order (Orthodox), the third with strategic social relations (Pragmatist), the fourth with progress (Reward) and the fifth with mustering resilience (Survivor). We proposed a syncretised measure of worldviews that synthesises the commonalities and serves the purposes of typification.

In this light, we proceeded to pose and examine two hypotheses. Firstly, we proposed that the Reward and Localised worldviews would endorse items proposing the legalisation of recreational cannabis whilst the Orthodox and Pragmatist worldviews would oppose. We reasoned that those individuals pursuing progress (Reward) would see in recreational cannabis an opportunity, whilst those individuals attuned to the needs of others (Localised) would empathise with those using cannabis for relief. Conversely, we reasoned that those individuals with firm convictions about a transcendental social order (Orthodox) would oppose the proposals for fear that getting high would conceal further the truths of revelation. Finally, we reasoned that individuals adopting a zero-sum worldview (Pragmatist) would suspect exploitative intent and react in their turn by opposing the proposals. Our expectations were partially supported. The Orthodox worldview stood in stark contrast with all others in opposing three of the four items we presented. In this way, the Orthodox worldview essentially constitutes the resistance to legalisation of recreational cannabis. This is understandable in light of the fact that the act of consuming cannabis for recreational purposes does not align with current laws, social rules, or practices of any organised faith in Western societies. These institutions more strongly advocate spiritual asceticism as a remedy to ails and strife than altered states of consciousness. Unlike the Orthodox type, the remaining four worldviews did not stand out in their endorsement or resistance to the proposals relative to any other worldview. This finding suggests that the policy proposals can be accommodated in the various worldviews and that they are neither perceived as necessarily utopic nor as unavoidably anomic, as is the case for the Orthodox type. We suggest that the resistance mounted by the Orthodox worldview is explored further through qualitative research to understand better its justificatory grounds.

In light of the above, it is worth noting that our study of worldviews fulfilled our original aim to identify coalitions (Koltko-Rivera, 2004; Buhagiar and Sammut, 2020) for and against the recreational cannabis issue. By studying worldviews, we were able to identify a characteristic outlook that marks the opposition. Whilst the predictive power of the worldview variables on their own is rather small in our study, it is worth bearing in mind that our measures isolated the effects of single worldviews on single items, as per our scholarly intent. Consequently, we could not aspire to observe high predictive power using such measures. Koltko-Rivera (2004) argues that the study of worldviews as a predictor variable is expected to increase *R*^2^ values by 5 to 10%. Our findings fell short of this criterion. Whilst our predictive models based on demographic and worldview variables ranged between 2 and 9%, the singular effect of the only worldview that exercised an influence in this particular case ranged from 1.1 to 1.8%. However, it is worth bearing in mind that Koltko-Rivera’s suggestion is based on dimensional measures, which are essentially continuous measures and therefore additive, rather than categorical measures which are not. We could have used the relative endorsement of the various categories as continuous variables and included all of them in our predictive models. This would have provided an insight into the overall proportion of variance accounted for by worldviews when all of them are considered simultaneously. However, this would not have enabled us to identify which particular worldview, if any, exercised an influence on this particular issue and which other worldview/s stood in association or opposition with it. Consequently, we deem the categorical exercise we pursued in this study as equally informative and we do not consider our below threshold *R*^2^ value a limitation. It is also worth noting that with regards to other policy issues or other controversial objects in social life (e.g., abortion in Malta), the proportion of variance accounted for might change and be higher or lower than the one we reported depending on which worldviews are influential and to what extent. We consider these issues to be empirical facts. We also maintain that the measurement of worldviews as categorical types remains useful in understanding alliances that are forged in the social order for or against particular issues, as in our present case. This aspiration is also underlined by Koltko-Rivera (2004) although an additive dimensional model would preclude such an understanding due to its additive properties. It is, however, clearly fulfilled in the present case. It is this methodological choice that helped reveal the characteristic influence of the Orthodox worldview on evaluating recreational cannabis proposals, as detailed above.

This being said, we contend that future research is needed to determine the further utility of studying the relative endorsement of different worldviews by the same subjects using continuous measures. These should prove particularly useful when examining interaction effects between different variables on the evaluation of particular proposals. It is also worth noting that, in our study, the proportion of variance accounted for by the worldviews measure on certain items was three times that of demographic effects combined (e.g., Item 4). Across the various items we administered, the effect of worldviews was approximately commensurate with the variance accounted for by demographic variables, which in our view is a notable finding. Future research is needed to determine whether particular worldviews are associated or interact with particular demographic categories. Such studies stand to yield much higher outputs in predictive power, as proposed by Koltko-Rivera (2004). Continuous worldview measures lend themselves better to the study of interaction effects than do categorical ones.

Be they dimensional or categorical types, we maintain that the study of worldviews when examining social issues is highly consequential. As detailed, it serves to reveal factions of support or opposition toward particular proposals in a given society. Worldviews shed light on the characteristic social groups who might reasonably coalesce to advance a particular outcome, which raises an interesting point regarding the relative distribution of worldviews in societies at particular points in time. This also helps explain why some issues meet stiff opposition initially only to be endorsed in the same society at a later point, or vice-versa. Shifting proportions of worldviews can account for such temporal dynamics. Additionally, the study of distributions of worldviews raises further interesting comparative questions regarding why some issues receive support in one setting but are rejected in another, as the proportional distribution of worldviews supporting or rejecting particular issues varies across jurisdictions. This was indeed a key finding for Salvatore et al. (2018) in their study of the distribution of symbolic universes across EU member states.

We find, therefore, that there is substantial merit in studying worldviews when it comes to the psychological study of political concerns. Buhagiar and Sammut (2020) argue that individuals coalesce around social representations that pursue particular projects. The current study demonstrates that worldviews enable processes of coalition building, by advocating plausible construals of novel objects or events that serve, or are aligned with, the prevalent order as perceived by the cognitively embedded subject. In this way, the study of worldviews answers the question of how social representations ‘stick’. Breakwell (2014) argues that stickiness is a feature of social representations, with which individuals interact. She notes that some social representations seem to have a greater tendency to attract adherents and that this is a function of the social representation itself as well as its processes of transmission. We argue, however, that stickiness is a function of the interlinkage mechanism between social representation on the one hand and individual cognition on the other. For an appeal to stick, the appeal must cognitively fit the individual receptor that is in force at the level of the subject. The individual receptor is what we understand to be the characteristic worldview the subject has adopted in an embedded manner to engage in social life. To use an analogy, stickiness operates like Velcro, by bringing together two elements that fit one another. In similar fashion, a proposal that fits a worldview (for or against) stands to stick whilst one that does not simply rebounds and leaves no discernible effect even if cognitively processed. In our study, the proposals for rejecting the legalisation of recreational cannabis stuck with those expressing an Orthodox worldview across a range of items. For others with different worldviews, the proposals were individually evaluated but demonstrated no consistency across the range or relative to others.

Future research is recommended to investigate how and when might individuals be inclined to shift their worldviews from one to another and to examine the consequences of such shifting. We contend that worldviews help individuals navigate the complexity of social life. Life circumstances may well challenge such predispositions and it will be interesting to find out in what manner do individuals cope with changing circumstances that require psychological adaptation. It is reasonable to expect more malleability in worldviews than other psychological inclinations, such as personality traits. On this note, future research is recommended to examine the links, if any, between personality traits and avowed worldviews. One the one hand, it is reasonable to expect that some traits are more inclined to adopt some worldviews over others. On the other hand, it is not unreasonable to expect individual differences amongst those sharing the same worldview given the latter’s presumed role in adaptation to life circumstances. With regards to the issue of recreational cannabis, we would like to note that our study is not an evaluation of any particular policy proposal. Nor is it meant to represent the entire population of Malta. It is very likely that other variables may be more predictive of support for recreational cannabis legislation, such as actual cannabis consumption. These variables, however, were beyond the scope of the present inquiry. Given the current findings, however, we recommend further research to examine whether cannabis use moderates the relationship between worldviews and support for cannabis legislation. Further research is also recommended to ascertain a fuller representation of the Maltese population including segments that were excluded from the current analysis, such as foreign nationals resident in Malta.

## Conclusion

The study of worldviews is consequential in understanding the political alliances achieved by interacting *like-minded* individuals, of which the present study is an example. Our research demonstrates that proposals for the legalisation of recreational cannabis are resisted chiefly by those subscribing to an Orthodox worldview. Such alliances enable the pursuit of collective projects that fulfil their worldview aspirations. In the relational process, social representations are construed in collective, self-serving ways (Buhagiar and Sammut, 2020). We propose that social representations stick when construals correspond with matching worldviews in a way that serves to preserve or repel the worldview’s prototypical social order. Consequently, we contend that the study of worldviews is key to understanding political behaviour and the formation of coalitions for action pursuing personal goals in social and political ways.

## Data Availability Statement

The raw data supporting the conclusions of this article will be made available by the authors, without undue reservation.

## Author Contributions

GS, RM, and NB contributed to conception and design of the study. RM piloted the questionnaire. GS performed the statistical analysis and wrote the first draft of the manuscript. All authors contributed to manuscript revision, read, and approved the submitted version.

## Conflict of Interest

The authors declare that the research was conducted in the absence of any commercial or financial relationships that could be construed as a potential conflict of interest.

## Publisher’s Note

All claims expressed in this article are solely those of the authors and do not necessarily represent those of their affiliated organizations, or those of the publisher, the editors and the reviewers. Any product that may be evaluated in this article, or claim that may be made by its manufacturer, is not guaranteed or endorsed by the publisher.

## Footnotes

1
www.yourmorals.org
